# *In vitro* digestion and human gut microbiota fermentation of *Bletilla striata* polysaccharides and oligosaccharides

**DOI:** 10.3389/fcimb.2023.1105335

**Published:** 2023-02-01

**Authors:** Qiqi Wang, Huimin Chen, Mingzhu Yin, Xue Cheng, Hui Xia, Haiming Hu, Junping Zheng, Zhigang Zhang, Hongtao Liu

**Affiliations:** College of Basic Medical Sciences, Hubei University of Chinese Medicine, Wuhan, China

**Keywords:** *Bletilla striata*, polysaccharides, oligosaccharides, gut microbiota, short-chain fatty acids, free radicals

## Abstract

**Background:**

*Bletilla striata* is one of the commonly used traditional Chinese medicine. *B. striata* polysaccharides (BP) and oligosaccharides (BO) are one of the main components of *B. striata*, which have been proved to have a variety of biological activities. However, the digestion and fermentation characteristics of BP and BO are still unclear.

**Methods:**

The study evaluated different prebiotic effects of BP and BO by *in vitro* simulating digestion and gut microbiota fermentation.

**Results:**

The results show that the simulating saliva partly degraded BP, but had no effect on BO. The molecular weights of BP and BO remained basically unchanged in gastric and intestinal digestion. In addition, BP and BO could be rapidly degraded and utilized by gut microbiota. During *in vitro* fermentation, the growth rates of the BP and BO groups were higher than that of the Control group and the pH value and total carbohydrate content in BP group and BO group decreased significantly. Although the reducing sugar level in the BO group decreased rapidly, it remained at a low level in the BP group. Both BP and BO improved the composition and structure of gut microbiota, indicative of the upregulated abundances of *Streptococcus* and *Veillonella*, and the downregulated populations of Escherichia and Bacteroides. There were differences in the SCFA production by gut microbiota and antioxidant activities between the BP and BO groups. The fermentation broth of the BP group displayed a stronger suppression of O_2_-, but a higher scavenging effect on DPPH for the BO group.

**Conclusions:**

BP and BO displayed different digestion and fermentation characteristics *in vitro* due to their distinct polymerization degrees. The study point towards the potential of BP and BO as prebiotics in the application to human diseases by selectively regulating gut microbiota in the future.

## 1 Introduction

*Bletilla striata* is a perennial herb of the genus *Bletilla* in Orchidaceae, and its dry tubers are commonly used in traditional Chinese medicine (He et al., 2017). About 261 compounds have been isolated from *B. striata*, mainly including benzenes, dihydrophenanthrene, diphenyl phenanthrene, phenanthrene, triterpenoids, and polysaccharides (Xu et al., 2019). Among them, *B. striata* polysaccharide (BP) is one of the main medicinal components of *B. striata*, and its content in dried tubers can reach up to 60% (Peng et al., 2014; Xu et al., 2019). BP is a neutral heteropolysaccharide mainly composed of glucose and mannose (Chen et al., 2021; Zhai et al., 2021). So far, BP has been widely used in the food and pharmaceutical industry as a thickener, lubricant, moisturizer, and emulsifier for its unique viscosity and self-degradability (Ji et al., 2020). In previous studies, BP displayed a variety of biological activities, such as promoting coagulation, anti-inflammation, anti-virus, anti-tumor, and anti-oxidation (Chen et al., 2018; Lai et al., 2018; Liu et al., 2019; Zhang et al., 2019a; Zhang et al., 2019b; Zhai et al., 2021).

Gut microbiota refers to all the microbial communities in the intestine, with a total number of cells above 10^14^. Firmicutes and Bacteroidetes are the main components of human gut microbiota (Lynch and Pedersen, 2016). As a kind of organic substance, prebiotics cannot be digested and absorbed by the human body. However, prebiotics may selectively promote the metabolism and proliferation of beneficial intestinal bacteria to improve host health (George Kerry et al., 2018; Hu et al., 2020; Cronin et al., 2021). Plant polysaccharides and their hydrolyzed oligosaccharides are essential components of prebiotics. According to the reports, gut microbiota contains about 130 glycoside hydrolase (GH) families, 22 polysaccharide lyase (PL) families, and 16 carbohydrate esterase families, which enable the gut microbiota to degrade various dietary polysaccharides (Guan et al., 2021; Zhang et al., 2021). Plant polysaccharides are not easy to be degraded in the upper digestive tract for the lack of degradation enzymes. On the contrary, in the colon, polysaccharides can be degraded to oligosaccharides and monosaccharides through different degradation systems or converted to beneficial metabolites like short-chain fatty acids (SCFAs) (Singh, 2019; Cronin et al., 2021). Then, these newly produced substances will be absorbed by the human body to regulate host physiological functions as calory intake or signaling molecules.

Studies show that BP could be degraded to *B. striata* oligosaccharide (BO) with appropriate polymerization degree and low molecular weight by acid hydrolysis. And BO had a particular therapeutic effect on obesity and non-alcoholic fatty liver disease by regulating gut microbiota and their metabolites (Hu et al., 2020; Hu et al., 2022). However, no studies show whether there are some differences in the prebiotic characteristics between BP and BO.

In this study, BP was obtained by water extraction and alcohol precipitation. BO with a certain degree of polymerization was prepared by acid hydrolysis of BP. *In vitro* digestion and fermentation models were used to study the digestion and fermentation process of saccharides. The regulatory effect of BP and BO on gut microbiota was verified by analyzing the fermentation products and their antioxidant abilities. Our studies were expected to provide evidence for the different prebiotic activities between BP and BO.

## 2 Materials and methods

### 2.1 Materials and reagents

Dry *B. striata* tubers were bought from Hubei Zexi Traditional Chinese Medicine Technology Co., Ltd. (Qichun, Hubei, China). The authenticity of medicinal materials was identified by Dr. Xiongjie Sun of Hubei University of Chinese Medicine. Vitamins, yeast extract, peptone, bacterial DNA extraction kit, bile salts, and L-cysteine were obtained from Beijing Solarbio Science & Technology Co., Ltd. (Beijing, China). Pepsin, α-amylase, pancreatin, trypsin, and SCFAs standards (Acetic acid, propionic acid, butyric acid, pentanoic acid, and indole) were bought from Shanghai Aladdin Biochemical Technology Co., Ltd (Shanghai, China). All materials were of standard analytical grades.

### 2.2 Preparation of BP and BO

Fresh and dry tubers of *B. striata* were ground and sifted through a 100-mesh sieve. *B. striata* power (30 g) was mixed with distilled water (1:40, w/v) and extracted at 90°C for 2 h (Xu et al., 2019). After centrifugation at 6000 ×g for 5 min, the supernatant was collected and concentrated in a rotary evaporator to 1/4 of the original volume. Next, anhydrous ethanol was slowly added to the supernatant in a ratio of 1:4. The precipitate was obtained after the solution was left standing at 4°C for 24 h. After the centrifugation at 5000 ×g for 5 min, the precipitate was collected and re-dissolved in distilled water. The protein was removed using a Sevage method (Wang et al., 2019; Liu et al., 2021). Finally, the aqueous phase was concentrated at 40°C followed by freeze-drying to obtain BP (Chen et al., 2018).

BO was prepared according to the reference (Hu et al., 2022). In brief, 1 g of BP was added to 100 mL distilled water and stirred at 90°C to dissolve. The solution reacted with 1.25 M of trifluoroacetic acid (v/v, 1:9) at 90°C for 1.5 h, and 10% ammonia (v/v) was added to terminate the reaction. The solution was concentrated to 1/5 of the original volume in a rotary evaporator and freeze-dried to obtain BO.

### 2.3 Characterization of BP and BO

#### 2.3.1 Chemical composition analysis

The total sugar content of BP and BO was measured using a phenol sulfuric acid method and was calibrated with a D-mannose standard (Dubois et al., 1956). The reducing sugar content was quantified using a 3, 5-dinitrosalicylic acid colorimetry method and calibrated with a D-mannose standard (Miller, 1959). The total proteins were detected by a Bradford method using the standard curve prepared with bovine serum albumin (Bradford, 1976). The uronic acid content was measured by a meta-hydroxybiphenyl method using glucuronic acid as the standard (Blumenkrantz and Asboe-Hansen, 1973).

#### 2.3.2 Molecular weight determination

The molecular weights of BP and BO were measured by a high-performance gel permeation chromatography (HPGPC) method (Chen et al., 2021). The analysis was performed on Waters1525 equipped with TSK gel GMPWXL column (7.8 mm × 300 mm). The column was eluted with ultrapure water at a flow rate of 0.6 mL/min at 30°C. Before analysis, the sample solution was prepared with ultrapure water and filtered through a 0.22 μm filter. The injection volume was 20 μL. Dextrans with different molecular weights (1−71 kDa, Sigma, MO, USA) were used as the standards to obtain the calibration curve.

#### 2.3.3 Monosaccharide composition analysis

The monosaccharide composition of BP and BO was determined using a PMP derivatization method (Fu et al., 2018; Chen et al., 2021). Briefly, 50 mg of saccharide sample was hydrolyzed with 3 mL of trifluoroacetic acid (2 M) at 120°C for 2 h. The excess trifluoroacetic acid was removed by evaporation under reduced pressure at 45°C. After that, 600 μL of NaOH (0.3 M) and 600 μL of PMP (0.5 M) were added to the sample solution. The mixture was incubated at 70°C for 30 min, and the mixture was neutralized with 0.3 M HCl and extracted with chloroform three times. The aqueous layer solution was filtered through a 0.22 μm filter before analysis.

The samples were analyzed with Uranus 5u C_18_ column (4.6 mm × 250 mm, 5 μm) at 30°C. The mobile phase was acetonitrile (A) and 0.02 M of ammonium acetate (B). The gradient elution conditions were: 0−5 min, 16% A; 5−17 min, 16%−19% A, 17−30 min; 19%–22% A; 30–35 min, 22%−16% A. The flow rate was 1 mL/min, and UV absorption was measured at 254 nm. The injection volume was 10 μL. Fucose, D-glucose, rhamnose, xylose, mannose, galactose, arabinose, glucuronic acid, and galacturonic acid were used as the standards.

#### 2.3.4 Methylation analysis of BP

The glycosidic bond composition of BP was analyzed by methylation reaction and GC-MS analysis (Chen et al., 2021). BP (5 mg) was dissolved in 2 mL of dimethyl sulfoxide and reacted with 50 mg NaH for 1 h. The solution was incubated with 1 mL CH_3_I at 25°C in the darkness for 2 h. Methanol was added to the solution to complete the reaction. After that, 5 mL H_2_O was added to terminate the reaction. After the extraction with chloroform, the methylated polysaccharide was subjected to acid hydrolysis, NaBH_4_ reduction, and acetic anhydride acetylation. The sample was finally extracted with CH_2_Cl_2_ for GC-MS analysis.

### 2.4 *In vitro* digestion of BP and BO

#### 2.4.1 *In vitro* saliva digestion

The simulated digestion in saliva was carried out according to the reported method (Huang et al., 2019; Huang et al., 2020). Simulated salivary fluid (SSF) was prepared by dissolving 0.0764 g/L NaCl, 0.0133 g/L CaCl_2_, and 0.1491 g/L KCl into distilled water. The pH of the solution was adjusted to 7.0 using 0.1 M of NaHCO_3_. BP or BO was dissolved in distilled water with a final concentration of 2.5 mg/mL. Then, 20.0 mL BP solution (BP group) or BO solution (BO group) was mixed with 20.0 mL SSF and 1 mL salivary α-amylase (1500 U/mL), followed by the incubation in a water bath (37°C). An equal volume of distilled water (without BP or BO dissolved in it) was used in the Control group. During the digestion, a 2.0 mL reaction mixture was withdrawn at 0, 5, 15, and 30 min, respectively. The collected mixture was boiled in a water bath for 5 min to deactivate salivary amylase. After the simulated salivary digestion, the reducing sugar content and molecular weights of BP and BO in the digestion solution were determined. The experiment was repeated three times.

#### 2.4.2 *In vitro* gastric digestion

The *in vitro* gastric digestion was performed as the described method with minor modification (Li et al., 2020). After the salivary digestion, the pH of the mixture was immediately adjusted to 3.0 by HCl (1 M) for the subsequent gastric digestion. In brief, 20 mL salivary digestion was mixed with 4.72 mg pepsin and 20 mL simulated gastric fluid (GSF) electrolyte solution (3.1 g/L of NaCl, 1.1 g/L of KCl, 0.6 g/L of NaHCO_3_, 0.15 g/L of CaCl_2_, pH 3.0), followed by the incubation in a water bath (37°C). During the digestion, a 2 mL reaction mixture was withdrawn at 0, 1.0, 3.0, and 5.0 h, respectively. The collected mixture was boiled in a water bath for 5 min to deactivate the pepsin enzyme. After the simulated gastric digestion, the reducing sugar content and molecular weights of BP and BO in the digestion solution were determined. The experiment was repeated three times.

#### 2.4.3 *In vitro* simulated intestinal fluid digestion

The *in vitro* simulated intestinal fluid digestion was performed as the described method with some modifications (Li et al., 2020; Ma et al., 2021). After the gastric digestion, the pH of the mixture was immediately adjusted to 7.0 using NaHCO_3_ solution (1 M) for the subsequent intestinal fluid digestion. In brief, the simulated intestinal fluid (SIF) was prepared by dissolving 5.4 g/L NaCl, 0.65 g/L KCl, and 0.33 g/L CaCl_2_ into distilled water, and the pH of the solution was adjusted to 7.0 by NaHCO_3_ solution (0.1 M). The intestinal fluid digestion was mixed with SIF-containing pancreatin solution (7%, w/v), bile salt solution (4%, w/v), and 26 mg trypsin at a ratio of 3:10 (v/v). Then, the solution was incubated at 37°C, and 2.0 mL of the mixture at different time points (0, 0.5, 1.0, 2.0, 4.0, and 6.0 h) was withdrawn and boiled in a water bath for 5 min to deactivate pancreatin enzymes. After the intestinal digestion, the reaction mixture was centrifuged, and the reducing sugar content in the supernatant was determined. Next, 80% (v/v) ethanol was used to precipitate BP and BO, and the molecular weights of both saccharides were determined. The experiment was repeated three times.

### 2.5 *In vitro* fermentation and subsequent broth analysis of BP and BO

#### 2.5.1 *In vitro* fermentation

The *in vitro* fermentation of fecal inoculum was conducted based on an established method with minor modification (Huang et al., 2020; Mao et al., 2021). Fecal samples were pooled from five healthy volunteers (three males and two females) who remained on a regular diet and did not use antibiotics within three months. The fresh fecal samples were mixed with sterilized PBS (including 0.5 g/L of L-cysteine) to obtain a 10% (w/v) fecal suspension.

The fermentation medium was prepared following the report (Ding et al., 2019; Huang et al., 2020). Briefly, 1 L basal medium consisted of 2.0 g peptone, 2.0 g yeast extract, 0.1 g NaCl, 0.01 g K_2_HPO_4_, 0.04 g KH_2_PO_4_, 0.01 g MgSO_4_·7H_2_O, 0.01 g CaCl_2_·2H_2_O, 2 g NaHCO_3_, 2 mL Tween-80, 0.05 g hemin, 10 μL vitamin K_1_, 0.5 g L-cysteine, 0.5 g bile salts, and 0.01 g resazurin. The basal medium was sterilized at 115°C for 20 min. Then, 1.0 mL fecal slurry was added to a 9.0 mL basal medium containing BP (2.5 mg/mL), BO (2.5 mg/mL), or not (Control group), respectively. The fermentation experiment was carried out in an anaerobic tank. All treatment groups were incubated with the fecal inoculum at 37°C in a thermostatic shaker. During the fermentation, samples were withdrawn at 0, 6, 12, 24, 36, and 48 h for further analysis. The experiment was repeated three times.

#### 2.5.2 Composition changes of fermentation broth

The OD_600_ of fermentation broth was detected with a multi-functional microplate reader at 600 nm. The pH value was measured using a pH meter. The contents of total carbohydrates and reducing sugars in fermentation broth at different time points were determined according to the methods as mentioned above.

#### 2.5.3 Gut microbiota analysis of fermentation broth

Gut microbiota analysis was performed as referenced (Hu et al., 2020). In brief, 2 mL of fermentation broth was collected after 24 hours of fermentation. The bacterial genomic DNA was extracted using an intestinal microbial DNA extraction kit. The DNA library was constructed by two-step PCR amplification, and the V3-V4 region of 16S rDNA was sequenced by Illumina miseq. After optimization, the sequence was analyzed by OTU cluster analysis, species diversity analysis, and species taxonomy analysis, as detailed in **Supplementary Methods**.

#### 2.5.4 Abundance quantification of gut microbiota at genus levels by qRT-PCR

The bacterial genomic DNA was extracted using an intestinal microbial DNA extraction kit (Solarbio, Beijing, China). In brief, the bacterial copies were measured by qRT-PCR using a SYBR QPCR mixture on a Bio-Rad CFX Connect Real-time system (Bio-Rad, CA, USA). The specific primer sequences were displayed in **Supplementary Table 6**. PCR mixture was initially heated at 95°C for 10 min, followed by 34 cycles of 95°C for 30 s, 55°C for 30 s, and 72°C for 30 s. Vectors containing specific bacterial 16S rDNA sequences were constructed as standards. The copies of specific bacteria in the fermentation broth were calculated according to the standard curves.

#### 2.5.5 Detection of short-chain fatty acids

After 24 hours of fermentation, the broth was centrifuged at 13,000 ×g for 5 min. The supernatant (0.5 mL) was evenly mixed with 1.0 mL pre-cooled methanol. After the low-temperature ultrasound for 10 min, the mixture was incubated overnight at 4°C. Next, the mixture was filtrated through a 0.22 μm membrane for the determination of SCFAs. Acetic acid, propionic acid, butyric acid, and valeric acid were used as SCFA standards. GC-MS was used to detect the SCFAs of all samples according to the reported method (Huang et al., 2020).

#### 2.5.6 Antioxidant activity of fermentation broth

The capacity of fermentation broth samples to scavenge 2,2-Diphenyl-1-picrylhydrazyl (DPPH) radicals were measured as referenced (Chen et al., 2021). The fermentation supernatant (200 μL), 0.2 mM DPPH (400 μL), and methanol (400 μL) were mixed and reacted at room temperature in the darkness for 20 min (Chen et al., 2021). The absorbance of samples was measured at 515 nm. With the basic medium as the control, the DPPH free radical scavenging activity was calculated according to the following formula: Scavenging rate (%) = (1 − As/Ac) × 100. Ac, absorbance of the control group; As, absorbance of the sample.

The capacity of fermentation broth samples to scavenge superoxide radicals was measured as referenced (Chen et al., 2021). The fermentation supernatant, 338 μM of nicotinamide adenine dinucleotide (NADH), 30 mM of phenazine methosulfate (PMS), and 72 μM of nitro blue tetrazolium chloride monohydrate (500 μL each) were mixed and reacted at room temperature for 15 min. The absorbance of samples was measured at 560 nm, and the essential medium was used as the control. The superoxide radical scavenging activity was calculated according to the following formula: Scavenging rate (%) = (1 − As/Ac) × 100. Ac, absorbance of the control group; As, absorbance of the sample.

### 2.6 Statistical analysis

All the experiments were performed in triplicate. The results were expressed as mean ± standard deviation (SD). GraphPad Prism (Version 7.0a, GraphPad Software Inc., San Diego, CA, USA) was used to analyze the results. Data were evaluated by one-way analysis of variance (ANOVA) with Duncan’s multiple range test. The value of *p* < 0.05 was considered statistically significant.

## 3 Results

### 3.1 Chemical composition of BP and BO

Based on previous studies (Wang et al., 2019; Chen et al., 2021), BP was prepared by water extraction and alcohol precipitation. Under the optimized conditions, the extraction rate of polysaccharides was 20%. The contents of total carbohydrates, reducing sugar and total proteins in BP were determined to be 78.69 ± 2.65%, 0.04 ± 0.05%, and 1.21 ± 0.70% (**Table 1**). The contents of reducing sugar and uronic acid were very low, indicating that BP was a neutral polysaccharide. Besides, BP was mainly composed of mannose (Man) and glucose (Glu), with a molar ratio of 76.19:23.81 (**Table 1**). As shown in **Supplementary Figure 1A**, the HPGPC chromatogram of BP was a relatively symmetrical peak, and the molecular weight of BP was calculated to be 221.17 kDa according to the peak time and standard curve (**Table 1**).

**Table 1:** Chemical and monosaccharide compositions of BP and BO.

Samples	BP	BO
Mw (Da)	221173	940
Chemical compositions		
Total carbohydrates (%)	78.69 ± 2.65	89.27 ± 1.84
Reducing sugar (%)	0.04 ± 0.05	43.68 ± 1.32
Protein (%)	1.21 ± 0.70	5.24 ± 1.35
Uronic acid (%)	5.28 ± 0.29	3.97 ± 0.46
Monosaccharide composition (molar ratio, %)		
Glucose	23.81	22.22
Mannose	76.19	77.78

The values are presented as mean ± SD (n = 3).

Next, the permethylated BP was hydrolyzed, reduced, and acetylated to provide the polymethacrylic acid (PMAAs), which was further analyzed by GC-MS. As shown in **Supplementary Figure 3A**, four signal peaks were identified in total ion chromatography, and their diagnostic fragments were indicated in **Supplementary Figures 3B−E**. Combining the retention time, the composition of methylated sugar, and peak fragments, we determined the methylated alditol acetates of BP (**Supplementary Table 1**). The mannose residues existed mainly as 1,4-linked-Man*p* and 1,6-linked-Man*p*. The glucosyl residues were presented as Terminal Glc*p* and 1,4-linked-Glc*p*.

The FTIR spectra analysis of BP was shown in **Supplementary Figure 4**. The bands at 893 cm^-1^ indicate the existence of β-glycosidic linkage in BP (Zhang et al., 2019a; Zhang et al., 2019b). The characteristic absorption peak at 956 cm^-1^ was known as the α-D-glucopyranose structure (Chen et al., 2021). The bands at approximately 875 cm^−1^ and 810 cm^−1^ contributed to the mannose residues (Kong et al., 2015; Qu et al., 2016).

BO was prepared by mild acid hydrolysis of BP. The contents of total carbohydrates, reducing sugar and proteins in BO were determined to be 89.27 ± 1.84%, 43.68 ± 1.32% and 5.24 ± 1.35% (**Table 1**). The mass spectrogram of BO showed that the polymerization degree of BO was 2−6 (**Supplementary Figure 5**). Similar to BP, BO was also composed of mannose and glucose with a molar ratio of 77.78:22.22 (**Table 1** and **Supplementary Figure 2**).

### 3.2 *In vitro* digestion changes of BP and BO

**Figure 1:**
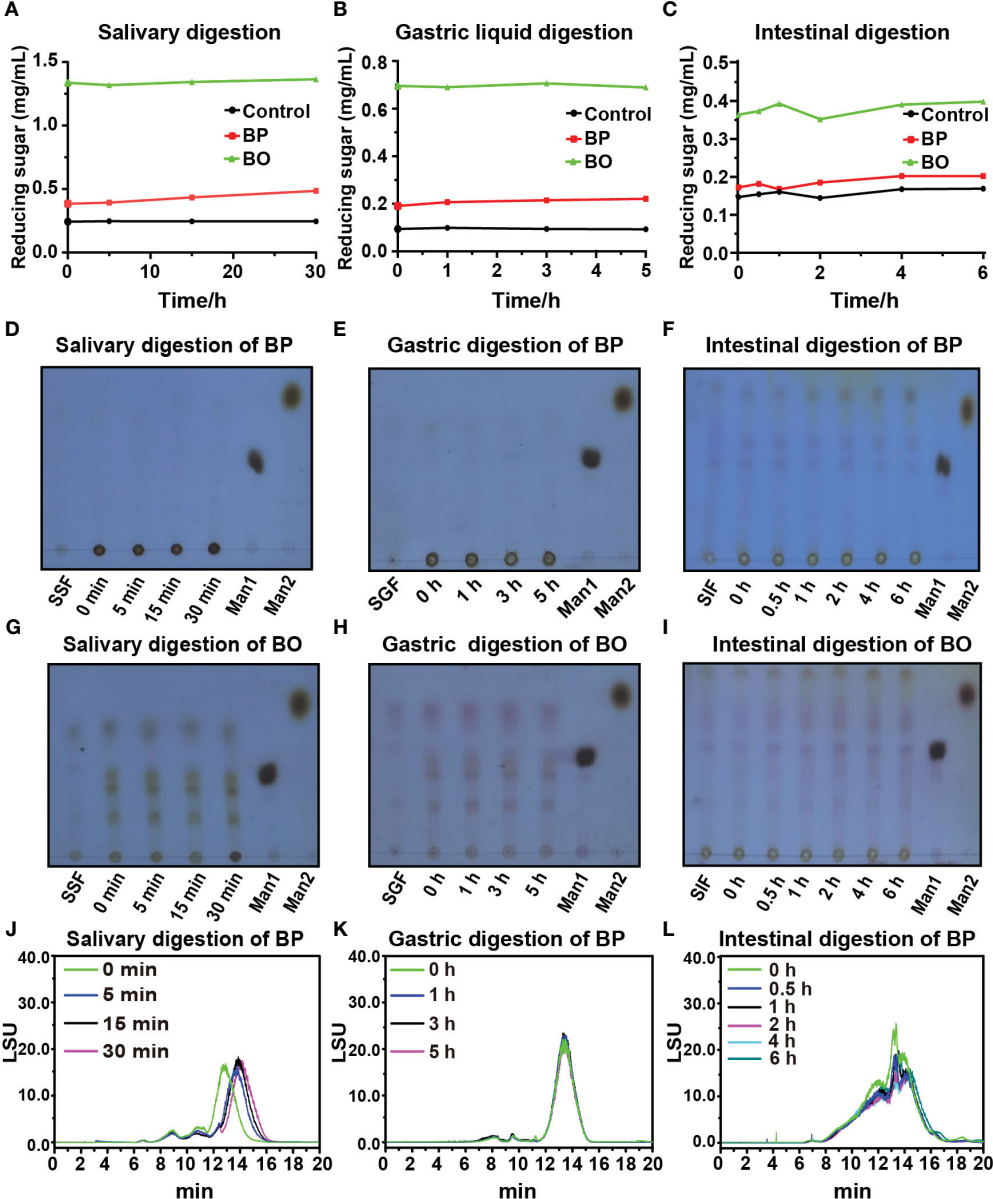
Changes in reducing sugar content and molecular weight of BP and BO during simulated saliva and gastrointestinal digestion. **(A−C)** Changes in reducing sugar content of BP and BO in simulated saliva and gastrointestinal digestion at different time points. **(D−F)** Thin layer chromatography (TLC) assay of BP that exhibited the changed molecular weights during simulated saliva and gastrointestinal digestion. **(G−I)** TLC assay of BO that exhibited the changed molecular weight during simulated saliva and gastrointestinal digestion. **(J−L)** HPGPC analysis of BP that exhibited the changed chromatogram during simulated saliva and gastrointestinal digestion. SSF, simulated salivary fermentation; SGF, simulated gastric fermentation; SIF, simulated intestinal fermentation; Man1:D-(+)-Mannose; Man2: α-1-6-Mannobiose. Data were presented as mean ± SD (n = 3).

Though some polysaccharides from food sources can be digested and utilized by the body directly through the digestive system, most plant polysaccharides will be fermented by gut microbiota to exert their biological activities before absorption (Cronin et al., 2021). In this study, the simulated digestion of BP and BO *in vitro* was carried out to explore whether both saccharides could be digested directly by the human body. The levels of reducing sugar in the simulated digestion process of BP and BO are shown in **Figures 1A−C**. There were no significant changes in the reducing sugar contents in the saliva, gastric juice, and intestine fluids. The results indicated that it was difficult for the human digestive enzymes to degrade BP and BO to produce reducing sugar. The thin layer chromatography (TLC) assay of simulated digestion products of BP was indicated in **Figures 1D−F**. There were no sugar bands with low molecular weights found in the digestion products of BP. The HPGPC analysis of simulated digestion products of BP was displayed in **Figures 1J−L**. As shown in **Figure 1J** and **Supplementary Table 2**, in the simulated saliva digestion, the molecular weight of BP gradually decreased to 21.03 kD with the increase in digestion time. In gastric liquid digestion, the molecular weight of BP was 21.89 kD at 0 h. After 5 h of digestion, the molecular weight of BP was 21.42 kD. And the molecular weight of BP was 19.97 kD after 6 hours of intestinal digestion. There was no significant change in the molecular weight of BP in gastric juice and intestinal fluid (**Figures 1K, L** and **Supplementary Table 2**). These results indicate that BP could be partly degraded by human digestive enzymes in saliva, but failed to be completely hydrolyzed to oligosaccharides or monosaccharides. BO is an oligosaccharide and the corresponding bands will appear on the silica gel plate. But the oligosaccharide bands of BO show no significant changes during three simulated digestion fluids (**Figures 1G−I**).

### 3.3 Effect of BP and BO on human gut microbiota by *in vitro* fermentation

#### 3.3.1 Changes of OD_600_ and pH during *in vitro* fermentation

**Figure 2:**
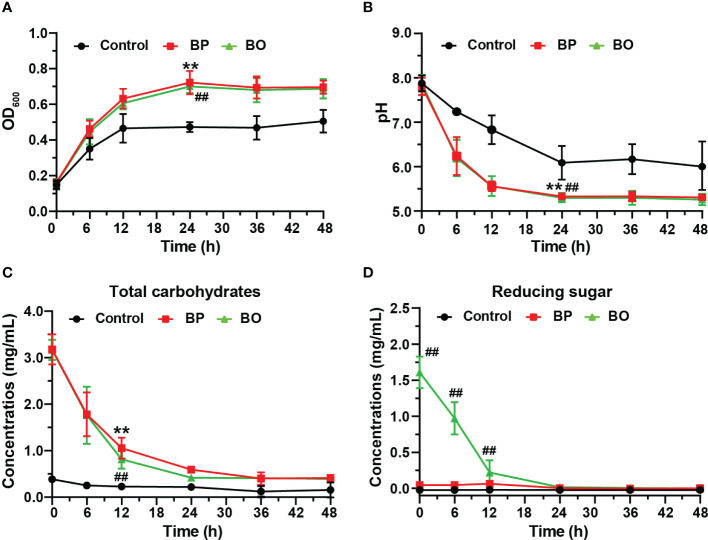
Changes of the growth curve, pH value, and fermentation broth composition during *in vitro* fermentation of BP and BO at different time points. **(A)** OD_600_ values of gut microbiota. **(B)** pH values of fermentation broth. **(C)** Total carbohydrate changes of fermentation broth. **(D)** Reducing sugar changes of fermentation broth. Data were presented as mean ± SD (n = 3). **p < 0.01, Control group vs. BP group; ^##^ *p* < 0.01, Control group vs. BO group.

The OD_600_ change reflected the growth of gut microbiota in bacterial media. As shown in **Figure 2A**, the OD_600_ value of the BP group increased from 0.16 ± 0.00 to 0.72 ± 0.07 during the first 24 hours, and that of the BO group raised from 0.16 ± 0.02 to 0.70 ± 0.04. For the Control group, the OD_600_ value raised from 0.15 ± 0.02 to 0.47 ± 0.03. The growth rates of the BP and BO groups in the logarithmic growth period were higher than that in the Control group. Also, the OD_600_ in the final platform period was significantly higher compared with the Control group (*p* < 0.05). The result shows that either BP or BO could improve the growth of gut microbiota.

The sugar products of bacteria metabolism can reduce the pH of culture media (Koh et al., 2016). As indicated in **Figure 2B**, the pH value of the BP group decreased from 7.81 ± 0.20 to 6.24 ± 0.43 after the fermentation for 6 h, and that of the BO group reduce from 7.88 ± 0.19 to 6.20 ± 0.41. The pH values of both groups were statistically lower than those of the Control group at all time points (*p* < 0.05), which decreased from 7.88 ± 0.18 to 7.24 ± 0.04. The reduction in pH values of the BP and BO groups may contribute to the rapid metabolite production of polysaccharides or oligosaccharides by gut microbiota (Huang et al., 2020).

#### 3.3.2 Changes of total carbohydrate and reducing sugar *in vitro* fermentation

Previous studies show that gut microbiota can degrade polysaccharides into reducing oligosaccharides and monosaccharides by secreting glycosidases (La Rosa et al., 2019; Singh, 2019). In this study, the fermentation of intestinal flora resulted in a decreased total carbohydrate and changed reducing sugar contents at different time points (**Figures 2C, D**). The concentration of total carbohydrates in the BP group decreased from 2.54 ± 0.30 mg/mL to 0.35 ± 0.08 mg/mL, but the concentration of reducing sugar had no significant change. For the BO group, the concentration of total carbohydrates decreased from 2.56 ± 0.23 mg/mL to 0.37 ± 0.01 mg/mL, and the content of reducing sugar reduced from 1.61 ± 0.22 mg/mL to 0.00 ± 0.02 mg/mL. There was no change in the concentration of either total carbohydrate or reducing sugar for the Control group during the whole fermentation process. Compared to the Control group, the concentrations of total carbohydrates in both the BP and BO groups decreased remarkably within the first 24-hour fermentation (*p* < 0.05). Though the content of the original reducing sugar in the BO group was much higher than that of the BP group (**Table 1**), it was consumed rapidly and almost fully utilized within 24 hours (**Figure 2D**).

#### 3.3.3 Changes in molecular weights of BP and BO during *in vitro* fermentation

**Figure 3:**
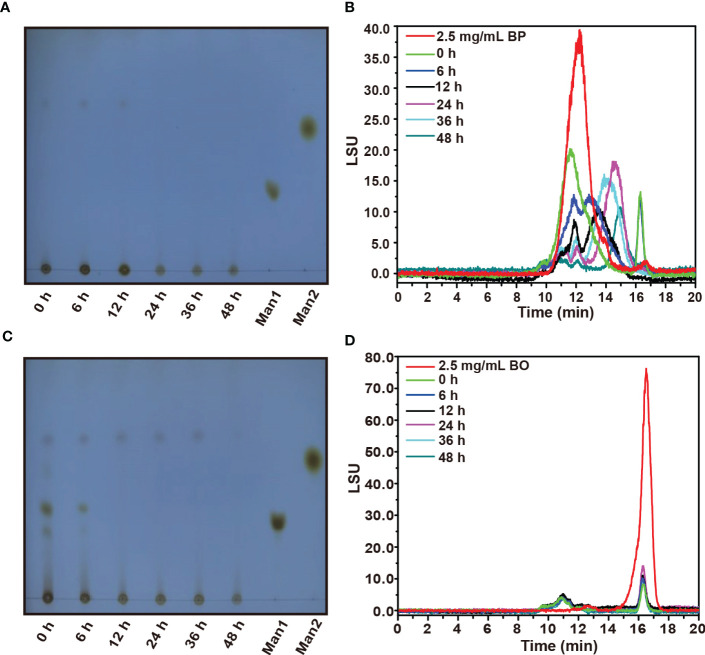
TLC and HPGPC analysis on degraded oligosaccharide products of BP and BO by gut microbiota during *in vitro* fermentation. **(A, B)** Molecular weight change of BP degradation products by TLC **(B)** and HPGPC **(A)** assay. **(C, D)** Molecular weight change of BO degradation products by TLC **(C)** and HPGPC **(D)** assay. Man1:D-(+)-Mannose; Man2: α-1-6-Mannobiose.

The TLC spectrum of BP fermentation products is shown in **Figure 3A**. During the fermentation, the imprint of BP at the original site was gradually weakened, but no new oligosaccharide bands appeared. We presume that the newly produced oligosaccharides may be rapidly utilized by gut microbiota, which was consistent with the change of reducing sugar in the fermentation broth of the BP group (**Figure 2D**). Based on the HPGPC analysis in **Figure 3B**, the average molecular weight of BP gradually decreased from 221.17 kDa to 3.00 kDa within 24 h. After the fermentation for 48 h, a new oligosaccharide peak was generated at 17 min, and its average molecular weight stabilized at about 3.00 kDa (**Supplementary Table 3**). The result suggests that gut microbiota preferentially chose low molecular weight carbohydrates as their substrates.

By TLC assay, the oligosaccharide bands of BO were observed with different polymerization degrees after the fermentation (**Figure 3C**). And these oligosaccharide products were quickly utilized by gut microbiota within 12 h as indicated by the weakened TLC detection band (**Figure 3C**). Due to the low polymerization degree of BO degradation products, we failed to obtain them by alcohol precipitation. Thus, the average molecular weight of BO in the culture medium did not change significantly during the fermentation (**Figure 3D** and **Supplementary Table 3**).

#### 3.3.4 Effects of BP and BO on composition of gut microbiota during *in vitro* fermentation

**Figure 4:**
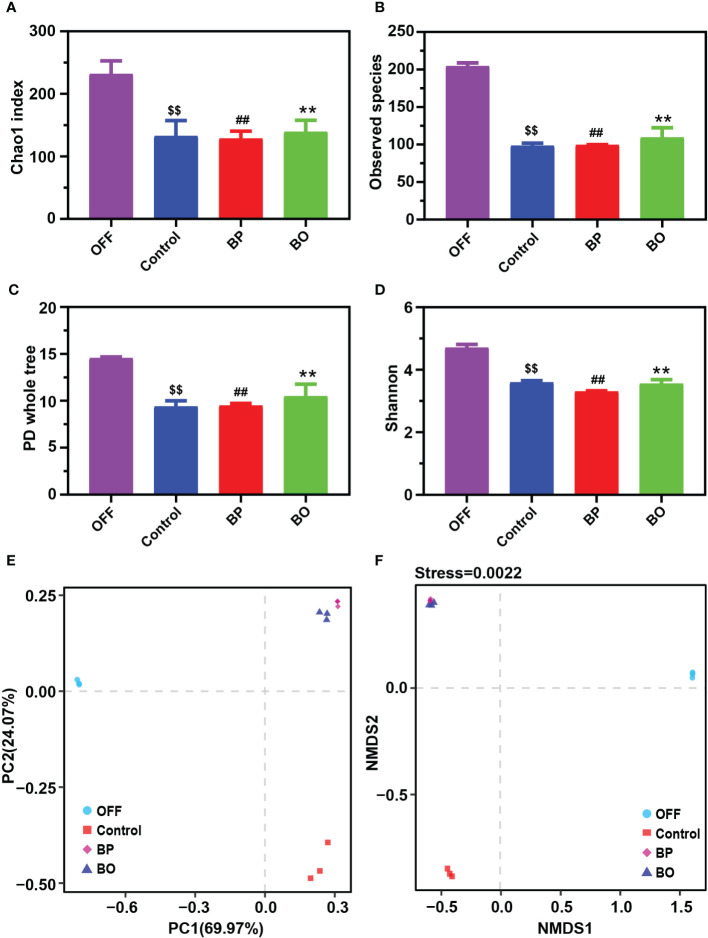
Effects of BP and BO on α-diversity and β-diversity of gut microbiota during *in vitro* fermentation. **(A–D)** α-diversity analysis of gut microbiota by Chao 1 index **(A)**, observed species **(B)**, PD whole tree **(C)**, and Shannon index **(D)**. **(E, F)** β-diversity analysis of gut microbiota by principal component analysis (PCA) and non-metric multidimensional scaling (NMDS) assay. OFF, Original fecal flora. Data were shown as mean ± SD (n = 3). ^$$^ *p* < 0.01, OFF group *vs*. Control group; ^##^ *p* < 0.01, OFF group vs. BP group; **p < 0.01, OFF group vs BO group.

In our previous studies, BO displayed a regulatory effect on the composition of gut microbiota in obese mice (Hu et al., 2020). In this study, 16S rDNA from human fecal fermentation samples were sequenced to verify the effects of BP and BO on the composition of gut microbiota. After removing the unqualified sequences, a total of 21,649 valid ones were obtained for each sample (**Supplementary Table 4**). The filtered sequences were clustered into an OTU with 97% similarity. The number of OTUs of each sample mainly represented the richness of sample diversity, and a total of 245 OTUs were obtained (**Supplementary Table 5**). The α diversity index of samples from different groups was indicated in **Figures 4A−D**. There were no marked differences in the α diversities of gut microbiota among the three experimental groups. However, they were significantly lower than that of the OFF (Original fecal flora) group (*p* < 0.05), similar to the results of previous *in vitro* fermentation studies (Chen et al., 2017; Chen et al., 2019; Zhou et al., 2020). The decreased gut microbiota diversity might be attributed to that the present *in vitro* culture system can’t entirely meet the growth conditions of all gut microbiota. The β diversity reflected the difference of species composition between different experimental groups. The results of PCA and NMDS (**Figures 4E, F**) suggested that both the BP and BO groups displayed a close cluster, which was away from the Control and OFF groups.

**Figure 5:**
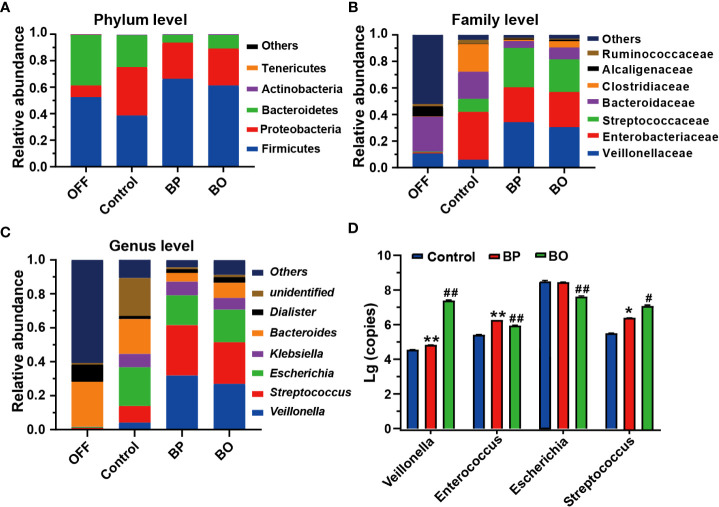
Bacterial taxonomic profiling of fermentation broth after different treatments. **(A)** Gut microbiota composition at the phylum level. **(B)** Gut microbiota composition at the family level. **(C)** Gut microbiota composition at the genus level. **(D)** Changes of bacteria copies at the genus level by qRT-PCR. Data were presented as mean ± SD (n = 3). *p < 0.05, **p < 0.01, Control group *vs.* BP group; *^#^p* < 0.05, ^##^ *p* < 0.01, Control group vs. BO group.

Next, we analyzed the composition and structure of gut microbiota among fermentation groups with different treatments by 16S rDNA sequencing (**Figure 5**). At the phylum level, the dominant bacterial communities in initial fecal samples of the OFF group were mainly Firmicutes, Bacteroidetes, and Proteobacteria (**Figure 5A**). And the relative abundances of Firmicutes, Bacteroidetes, and Proteobacteria reached 99.28% of the total bacterial community. After the fermentation for 24 h, the gut microbiota composition among the three groups was significantly changed. Compared to the Control group, the abundance of Bacteroides in the BP and BO groups was statistically reduced (38.11% for the Control group, 5.92% for the BP group, and 10.21% for the BO group). In comparison, the content of Firmicutes was significantly elevated (52.38% for the Control group, 66.26% for the BP group, and 61.20% for the BP group). The result indicated that both BP and BO treatment could regulate the composition of gut microbiota during fermentation. At the family level, BO and BP increased the abundances of Veillonellaceae and Streptococcaceae, but reduced the contents of Enterobacteriaceae and Bacteroidaceae. It seemed that BP displayed a stronger regulatory effect on gut microbiota communities than BO.

At the genus level, the change trends of gut microbiota after BP and BO treatment were similar to the above results (**Figure 5C** and **Supplementary Figure 7**). Overall, BP had a more significant impact on intestinal flora than BO. For instance, BP presented a more substantial effect on increasing the abundances of *Veillonella* and *Streptococcus*, and decreasing the contents of *Escherichia* and *Bacteroides* in comparison with those by BO treatment. Further, we confirmed the sequencing results of gut microbiota by qRT-PCR (**Figure 5D** and **Supplementary Figure 7**). In parallel, BP and BO greatly promoted *Veillonella* and *Streptococcus*, and suppressed *Escherichia*.

**Figure 6:**
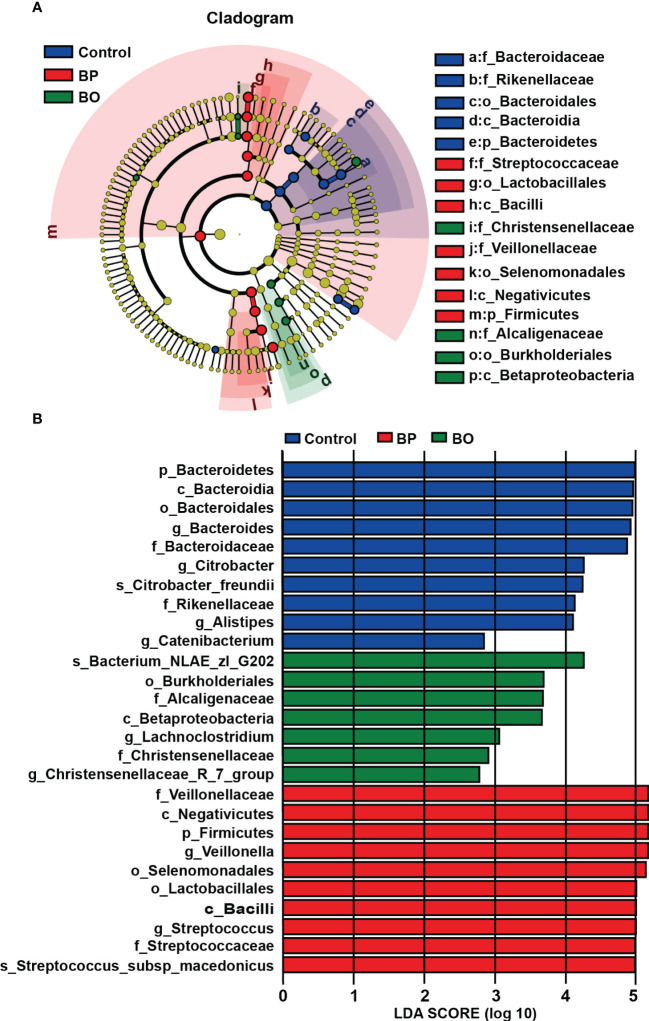
Identification of most characteristic taxa among three experimental groups by linear discriminant analysis (LDA) effect size (LEfSe). **(A)** Taxonomic cladogram obtained from LEfSe analysis on gut microbiota in individual experimental groups. **(B)** LDA scores calculated for taxa differentiation among experimental groups.

Linear discriminant analysis effect size (LEfSe) analysis is an algorithm for high-dimensional biomarker discovery and interpretation, which can find statistically different biomarkers between different groups. To identify the specific bacterial taxa among three groups with different treatments, we compared their gut microbiota compositions using the LEfSe method (**Figure 6**). The cladogram in **Figure 6A** revealed those taxa enriched in each group (‘p_’, phylum; ‘o_’, order; ‘c_’, class; ‘f_’, family). The results of LEfSe analysis are shown in **Figure 6A**. In the control group, BP group, and BO group, there were five, seven, and four dominant taxa with statistical significance identified in the Control, BP and BO groups, respectively. Then, linear discriminant analysis (LDA) coupled with LEfSe screened the significantly different taxa in individual experimental groups. As indicated in **Figure 6B**, ten, ten, and seven taxa with LDA scores higher than 2.0 were separate from the Control, BP, and BO groups. Among these bacteria, some belong to the probiotic ones. For example, Streptococcaceae, Lactobacillales, and Bacilli were the dominant characteristic bacteria in the BP group, while Christensenellaceae, Alcaligenaceae, Burkholderiales, and Betaproteobacteria dominated the BO group.

#### 3.3.5 Effects of BP and BO on SCFA levels produced by gut microbiota during *in vitro* fermentation

**Figure 7:**
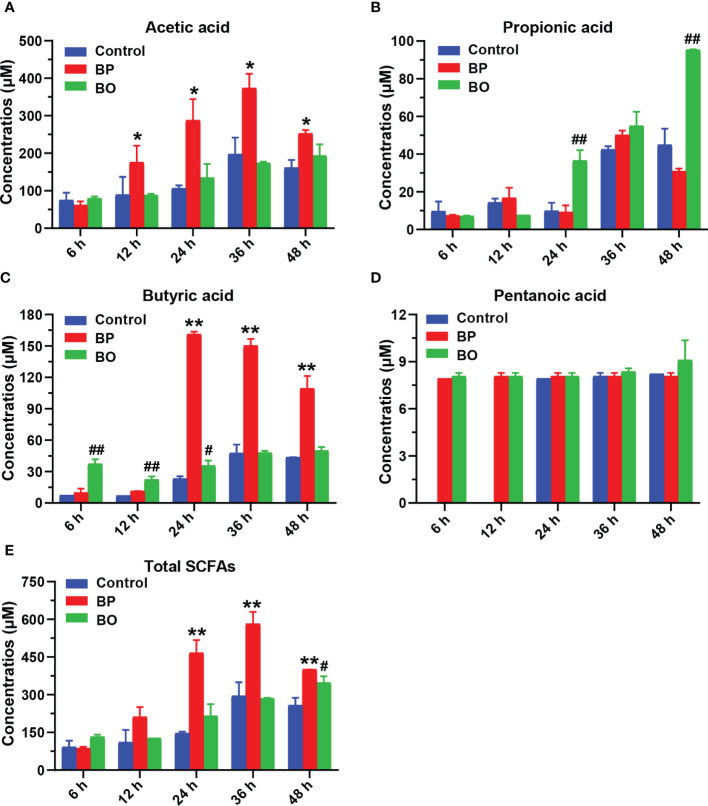
Concentrations of short-chain fatty acids (SCFAs) in culture medium after gut microbiota fermentation for 0, 6, 12, 24, 36, and 48 h. **(A–E)** Concentrations of Acetic acid **(A)**, Propionic acid **(B)**, Butyric acid **(C)**, Pentanoic acid **(D)**, and Total SCFAS **(E)** in the culture medium during the fermentation period. Data were presented as mean ± SD (n = 3). *p < 0.05, **p < 0.01, Control group vs. BP group; ^#^ *p* < 0.05, ^##^ *p* < 0.01, Control group vs. BO group.

As shown in **Figure 7** and **Table 2**, the concentrations of SCFAs in the culture medium were changed during *in vitro* fermentation of gut microbiota with BP or BO treatment. Within 24 hours, the acetic acid content of the BP group increased from 61.45 ± 9.90 μM to 287.76 ± 56.52 μM and from 11.41 ± 0.24 μM to 161.39 ± 2.41 μM for butyric acid (**Table 1** and **Figures 7A, C**). The concentrations of both SCFAs were significantly higher than those of the Control group (*p* < 0.05 or 0.01). For the BO group, the concentration of propionic acid in the culture medium increased from 7.09 ± 0.29 μM to a maximum of 95.37 ± 0.29 μM at 48 h (**Table 1** and **Figure 7B**) (*p* < 0.01, *vs.* Control group). Compared to the Control and BO groups, the gut microbiota of the BP group produced a more considerable amount of total SCFAs during fermentation (*p* < 0.01). Based on the above, although BO was easier to be utilized by gut microbiota, BP had more advantages in promoting SCFA production during *in vitro* fermentation.

**Table 2:** Concentrations of short-chain fatty acids (SCFAs) in culture medium after gut microbiota fermentation for 0, 6, 12, 24, 36, and 48 h.

Sample	Fermentation time (h)	SCFAs (μM)				
		Acetic acid	Propionic acid	Butyric acid	Pentanoic acid	Total
^1^Control	6	74.94 ± 19.78	9.72 ± 5.15	7.49 ± 0.00	–	92.15 ± 24.94
	12	89.18 ± 47.69	14.38 ± 2.00	7.15 ± 0.00	−	110.70 ± 49.69
	24	105.91 ± 8.48	9.92 ± 4.30	23.15 ± 2.41	7.93 ± 0.00	146.92 ± 6.59
	36	197.34 ± 44.51	42.52 ± 1.72	47.67 ± 8.19	8.08 ± 0.208	295.60 ± 54.21
	48	161.615 ± 20.14	44.95 ± 8.59	43.75 ± 0.24	8.23 ± 0.00	258.54 ± 28.67
^1^BP	6	61.45 ± 9.90	7.49 ± 0.29	10.04 ± 3.61^c^	7.931 ± 0.00	86.92 ± 5.99
	12	175.85 ± 44.51^ac^	16.81 ± 5.44	11.41 ± 0.24^c^	8.08 ± 0.21	212.14 ± 39.10
	24	287.76 ± 56.52^ac^	9.31 ± 3.44^c^	161.39 ± 2.41^ac^	8.08 ± 0.21	466.54 ± 50.89^ac^
	36	373.36 ± 38.62^ac^	50.22 ± 2.29	150.32 ± 6.50^ac^	8.08 ± 0.21	581.97 ± 47.62^ac^
	48	252.54 ± 9.54^a^	30.98 ± 1.43^c^	109.47 ± 11.80^ac^	8.08 ± 0.21	401.06 ± 0.62^a^
^1^BO	6	79.93 ± 2.24	7.09 ± 0.29	37.45 ± 4.33^bc^	8.08 ± 0.21	132.55 ± 8.65
	12	88.68 ± 3.18^c^	7.69 ± 0.00	22.47 ± 2.89^bc^	8.08 ± 0.21	126.92 ± 0.50
	24	135.39 ± 36.03^c^	36.65 ± 5.44^bc^	35.75 ± 4.82^bc^	8.08 ± 0.21	215.87 ± 46.50^c^
	36	174.11 ± 3.18^c^	55.08 ± 7.45	48.01 ± 1.93^c^	8.37 ± 0.21	285.56 ± 2.55^c^
	48	193.59 ± 30.03	95.37 ± 0.29^bc^	50.05 ± 3.37^c^	9.11 ± 1.25	348.12 ± 25.12^b^

Mean values in the same column with diﬀerent letters show signiﬁcant diﬀerences between diﬀerent treatments and diﬀerent treatment time, respectively (n = 3, *p* < 0.05). ^a^, mean value in the Control group and BP group shows statistical diﬀerence (n = 3, *p* < 0.05). ^b^, mean value in the Control group and BO group shows statistical diﬀerence (n = 3, *p* < 0.05). ^c^, mean value in the BP group and BO group shows statistical diﬀerence (n = 3, *p* < 0.05).

^1^ Control: negative control (no additional carbon source supplement); BP: experimental group (BP supplement); BO: experimental group (BO supplement).

^2^ −: not detected.

#### 3.3.6 Antioxidant activity analysis on fermentation broth of BP and BO group

**Figure 8:**
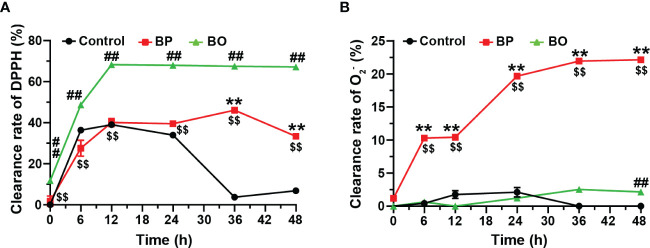
Scavenging effects of fermentation broth in BP and BO groups on DPPH and superoxide anion (O_2_-) free radicals. **(A)** Clearance rate of DPPH by BP or BO fermentation broth. **(B)** Clearance rate of O_2_- by BP or BO fermentation broth. Data were presented as mean ± SD (n = 3). **p < 0.01, Control group vs. BP group; ^##^ *p* < 0.01, Control group vs. BO group; ^$$^ *p* < 0.01, BP group vs.. BO group.

Finally, we detected the scavenging effect of fermentation broth on free radicals in the BP and BO groups. After the fermentation for 12 h, the scavenging capacity against DPPH free radical increased to 68.27 ± 0.27% for the BO group, 40.13 ± 1.95% for the BP group, and 39.06 ± 0.04% for the Control group (**Figure 8A**). During the whole fermentation period, the BO group displayed higher scavenging effect on DPPH (*p* < 0.01, vs. Control and BP groups). Only after the fermentation for 24 h, the BP group shows a better suppressive activity for DPPH than that of the Control group (*p* < 0.01). On the contrary, the BP group had a much higher scavenging effect on O_2_- than that of either the BO or Control group (*p* < 0.01), both of which failed to scavenge the production of O_2_- (**Figure 8B**). The maximal activity of BP was reached at 48 h (22.16 ± 0.16%). The results implied that glucomannans with different polymerization degrees had specific selectivity for the scavenging of free radicals.

## 4 Discussion

Studies show that the molecular weight of BP was between 7−40 kDa, which depends on the origin of *B. striata* and the extraction methods of BP (Wang et al., 2019; Chen et al., 2021). In keeping with these reports, the average molecular weight of BP in our studies was identified to be 18 kDa, mainly composed of 1,4-β-Mannopyranose, 1,4-β-glucopyranose, and a small amount of 1,4-α-glucopyranose (**Table 1** and **Supplementary Figures 1−4**). By the simulated saliva digestion, BP was gradually degraded with the extension of digestion time (**Figure 1J**). The main digestive enzyme in saliva is salivary amylase, which can break the α-1,4 polyglucoside bond of polysaccharides. And we did not detect the production of oligosaccharides and reducing sugar in salivary digestive juice (**Figures 1A, D, J**). This result suggests that saliva only degraded α-1,4 polyglucoside bonds, but not 1,4-β-polyglucoside bonds in BP (**Supplementary Figure 6A**). Since the digestive effect of human saliva on BP was relatively limited, it failed to produce the carbohydrates that could be directly used by the human body (**Figures 1A, D**). In the preparation of BO, α-1,4 polyglucoside bonds had been degraded by hydrolases (Data not shown) (Hu et al., 2020), so BO could not be degraded by the salivary digestive enzyme (**Figure 1J**). As for the simulated gastric and intestinal digestion, both had no effect on the molecular weights and reducing sugar contents of BP and BO (**Figure 1**). Previous studies also found that plant polysaccharides had good stability in gastric and intestinal juice (Chen et al., 2018). Based on the above, BP and BO could not be directly and completely digested in the upper gastrointestinal tract of humans, making them potential prebiotics for human health.

Gut microbiota plays a crucial role in host energy metabolism and immune system development (Lynch and Pedersen, 2016; George Kerry et al., 2018). The nutrition of intestinal microorganisms mainly comes from carbohydrates in the host diet, in which natural non-digestible polysaccharides are favorable substrates for improving the intestinal ecosystem Hu et al., 2020). For the utilization of carbohydrates, the gut microbiota has evolved a precise, variable, and complex carbohydrate utilization system to sense, capture and utilize polysaccharides (Tang et al., 2019; Cronin et al., 2021). For example, gut microbiota can synthesize a variety of glycosidases to decompose and ferment non-digestible polysaccharides, accompanied by changes of pH and total sugar content in the intestine (Huang et al., 2020). In this study, the pH value in the fermentation broth of three experimental groups gradually decreased, which may be due to the newly produced SCFAs by gut microbiota (**Figure 7**). Notably, during fermentation, the reducing sugar level in the BO group decreased rapidly. Still, it remained at a low level in the BP group (**Figure 2D**). It was reported that glycosidases secreted by intestinal strains only degraded mannan into oligosaccharides rather than monosaccharides, and the oligosaccharides were then transported into bacterial cells for degradation and utilization (La Rosa et al., 2019; Lindstad et al., 2021). This might be the reason why the concentration of total sugar decreased in the BP group during fermentation (**Figure 2C**), whereas the content of reducing sugar had no changes (**Figure 2D**). It was confirmed by TLC and HPGPC analysis (**Figures 3A, B**), in which the gut microbiota could only degrade BP to oligosaccharides with an average molecular weight of 3 kDa. We speculate that the subsequent degradation of oligosaccharides should occur in the cell wall or cytoplasm of intestinal bacteria, and this is required to be proved in future work.

Glucomannan has an excellent regulatory effect on gut microbiota. As a representative of glucomannan, Konjac polysaccharides (KGM) are composed of D-glucose and D-mannose, a similar structure to BP. It was reported that KGM could significantly increase the proportion of Firmicutes in gut microbiota while reducing the abundances of Proteus and Bacteroidetes (Mao et al., 2021; Song et al., 2021), consistent with our studies (**Figure 5A**). By 16S rDNA sequencing and qRT-PCR assay, we found that both BO and BP evidently increased the proportions of *Veillonella* and *Streptococcus* genera (**Figures 5C, D**). The two bacteria are normal flora in the human intestine (Liu et al., 2020). The utilization of carbohydrates and the tolerance to an acidic environment of *Streptococcus* have attracted much attention in recent years. In the presence of excessive sugars, *Streptococcus* can produce a large amount of lactic acid and promote the acidification of intestinal microenvironment under anaerobic conditions (Abranches et al., 2018). *Veillonella* is a gram-negative anaerobic bacterium with a weak ability to utilize polysaccharides. However, it can use short-chain organic acids as an energy source to convert them into acetic acid and propionic acid (Liu et al., 2020). When *Streptococcus* and *Veillonella* were co-cultured, *Veillonella* promoted the expression and secretion of *Streptococcus* glycosidase, and enhanced the ability of *Streptococcus* to degrade carbohydrates (Kara et al., 2006). In this study, *Streptococcus* and *Veillonella* were likely to cooperate in hydrolyzing BP and BO, increasing their proportions of gut microbiota and thus promoting the production of SCFAs during fermentation.

SCFAs are organic fatty acids composed of 1−6 carbon atoms, including acetic acid, propionic acid, butyric acid, and pentanoic acid (Koh et al., 2016). As the major products of dietary fiber fermented by gut microbiota in the colon, SCFAs can not only be used as substrates for the synthesis of sugars or lipids, but also regulate metabolic responses of the body as signaling molecules (Koh et al., 2016). SCFAs also improve intestinal health by regulating gut microbiota communities, maintaining intestinal barrier integrity, and preventing inflammation (He et al., 2020). Studies showed that the monosaccharide composition of polysaccharides had a great impact on the production of SCFAs. For example, the fermentations of galactose, mannose, and galacturonic acid in carbohydrates produced more butyric acid. And the production of propionic acid was predominantly due to the metabolism of glucose, xylose, mannose, and arabinose (Yang et al., 2013; Koh et al., 2016). In this study, there was a difference between BP and BO in promoting the formation of SCFAs. BP significantly promoted the formation of acetic acid and butyric acid, and BO enhanced the production of propionic acid (**Figure 7**). Considering that BP and BO have similar monosaccharide compositions and glycosidic bond types, their different polymerization degrees might be the critical reason for the distinct regulatory effects on SCFA production. Additionally, after the fermentation for 24 h, the content of total SCFAs in the BP group was the highest among the three experimental groups, suggesting that the regulatory effect of BP on gut microbiota was more lasting and stable than BO.

Excessive production of free radicals by oxidative stress was a fundamental reason for metabolic diseases (Dziabowska-Grabias et al., 2021). The antioxidative activity has been proven to be an essential physiological function of dietary fibers. There were few studies on the antioxidation changes of polysaccharides during gut microbiota fermentation. In this study, the BO group had a high scavenging effect on DPPH, while the BP group displayed a strong suppression of O_2_- (**Figure 8**). Different monosaccharide compositions, chain arrangements, chemical structures, and molecular weights may lead to variations in the antioxidant activity of polysaccharides (H. Chen et al., 2021). The results showed that the fermentation of BP by gut microbiota changed the molecular weight and spatial structure and produced many metabolites. Hence, the BP group had a much higher scavenging effect on O_2-_ than the BO or Control group. Since BP and BO were transformed into different products during gut microbiota fermentation, the antioxidant activities of their fermentation products may also have evident selectivity against the types of free radicals.

## 5 Conclusion

There were some differences between BP and BO in the process of digestion and fermentation *in vitro*. Although the polymerization degree of BP in simulated saliva was partly reduced, both BP and BO could not be degraded to oligosaccharides or monosaccharides by human digestive enzymes. BP and BO could be degraded and utilized by gut microbiota. During *in vitro* fermentation, the content of reducing sugar in the BO group decreased rapidly, but it remained at a low level in the BP group. Both BP and BO significantly affected the composition and structure of gut microbiota. Besides, BP and BO promoted the production of SCFAs by gut microbiota. BP had a better promotive effect on the formation of acetic acid and butyric acid, and BO mainly elevated the content of propionic acid. Finally, the fermentation broth of the BP group displayed a more robust suppression of O_2_-, but a higher scavenging effect on DPPH for the BO group. In summary, BP and BO were demonstrated to be potential prebiotics for human health, and their different polymerization degrees gave them pronounced selectivity in regulating gut microbiota.

## Data availability statement

The original contributions presented in the study are included in the article/**Supplementary Material**. Further inquiries can be directed to the corresponding authors.

## Author contributions

HL: Conceptualization, Supervision, Writing original draft, Funding acquisition. ZZ: Funding acquisition, Project administration, Supervision. QW: Methodology, Data curation, Resources, Writing original draft. HC: Writing original draft, Data curation, Formal analysis. MY: Data curation, Formal analysis. XC: Formal analysis, Software, Methodology. HX: Investigation, Writing original draft, HH: Formal analysis, Visualization, Methodology. JZ: Validation, Visualization. All authors contributed to the article and approved the submitted version.
