# Supplementary material for: In vitro digestion and human gut microbiota fermentation of Bletilla striata polysaccharides and oligosaccharides
Source: Front Cell Infect Microbiol. 2023 Feb 1;13:1105335. doi: 10.3389/fcimb.2023.1105335 (PMC9929950; doi:10.3389/fcimb.2023.1105335)
Supplement: Supplementary file 1 [file DataSheet_1.docx]

- *Bletilla striata* polysaccharides (BP) and oligosaccharides (BO) could not be degraded to monosaccharides by human digestive enzymes.
- BP and BO could be degraded and utilized by gut microbiota.
- BP and BO affected composition and structure of gut microbiota by *in vitro* fermentation.
- BP promoted production of acetic acid and butyric acid, and BO enhanced level of propionic acid by gut microbiota.
- BP fermentation broth displayed a strong suppression of O^2^-, but a higher scavenging effect on DPPH for BO group.
