# Supplementary material for: In vitro digestion and human gut microbiota fermentation of Bletilla striata polysaccharides and oligosaccharides
Source: Front Cell Infect Microbiol. 2023 Feb 1;13:1105335. doi: 10.3389/fcimb.2023.1105335 (PMC9929950; doi:10.3389/fcimb.2023.1105335)
Supplement: Supplementary file 2 [file DataSheet_2.docx]

**Supplementary information**

**Supplementary Methods**

1. Identification of polymerization degree of BO

2. 16S rDNA gene sequencing and analysis

3. Fourier transform infrared spectroscopy (FTIR) of BP

**Supplementary Figures**

Fig. 1. Chromatographic analysis of BP

Fig. 2. Chromatographic analysis of BO by HPLC

Fig. 3. GC-MS profile of methylated alditol acetates of BP

Fig. 4. FTIR spectra analysis of BP

Fig. 5. Structure analysis of BO

Fig. 6. Predicted structures of BP (A) and BO (B)

Fig. 7. Heatmap analysis of gut microbiota at genus level using LEfSe analysis

**Supplementary Tables**

Table 1. Sugar residue linkages of BP by methylation and GC-MS

Table 2. HPGPC analysis exhibiting molecular weight (MW) changes of BP and BO during in vitro digestion

Table 3. HPGPC analysis exhibiting molecular weight (MW) changes of BP and BO during in vitro fermentation

Table 4. Statistics on raw/clean reads by 16S rDNA sequencing

Table 5. Number of OUTs for single sample

Table 6. List of primer sequences for qPCR analysis of fecal samples

**Supplementary Methods**

1. Identification of polymerization degree of BO

To identify the polymerization degree of BO, HPLC-MS analysis was performed on an ultra-high performance liquid chromatography system (SCIEX, Framingham, MA, USA) with an Agilent Poroshell Hilic colum (2.1 × 100 mm, 2.7 μm) (Santa Clara, CA, USA) fitted with a Triple TOF 4600 high resolution mass spectrometry system (SCIEX, Framingham, MA, USA). The mobile phase was composed of 0.1% aqueous formic acid (A) and acetonitrile (B). The gradient elution was performed as follows: 0−1 min, 100% B; 1−2 min, 100% to 85% B; 2−32 min, 85% to 50% B; 32-33 min, 50% to 20% B; 33-37 min, 20% to 85% B; 37−44 min, 85% B. The injection volume was 10 μL and the flow rate was 0.03 mL/min. The ESI source in positive ion mode was used with resolving power 30,000 and a scan range of m/z 100-1000. The parameters were set as followed: capillary temperature, 600 °C; ion spray voltage, 5.5 kV in positive ion mode; sheath and auxiliary gas flow rate, 55 psi; collision energy, 35 eV.

1. 16S rDNA gene sequencing and analysis

For the genomic DNA of fecal samples, a pair of specific primers were fused into barcodes of sample DNA to amplify the V3-V4 hypervariable region of bacterial 16S rDNA (Forward: 5′-ACTCCTACGG GAGGCAGCAG-3′; Reverse: 5′-GACTACHVGGGTWTCTAAT-3′). PCR mixture was initially heated at 95 °C for 5 min, followed by 20 cycles of 95 °C for 30 s, 55 °C for 30 s, and 72 °C for 30 s. Reactions were completed at 72 °C for 10 min. Amplicons were then purified and pooled for pair-end sequence (2 × 300) following the standard protocol on an Illumina MiSeq platform (Illumina, San Diego, CA, USA) by Beijing Allwegene Tech (Beijing, China). The high-quality sequences were primarily generated using QIIME package (Quantitative Insights into Microbial Ecology, USA) (Version 1.8.0 <http://qiime.org/>). Sequences were chosen as followed: (1) average quality score >20 in 50 bp sliding window; (2) no less than 120 nucleotides; (3) sequences with overlap at least 10 bp; (4) complete match with primers; (5) distinct characters. All sequences were clustered into operational taxonomic units (OTUs) according to 97% similarity using UCLUST (Version 1.2.22 http:// www.drive5.com/uclust/downloads1_2_22q.html). Chimeric sequences were identified and discarded using Usearch (Version 8.1.1861 http://www.drive5.com/usearch/). The taxonomy of each 16S rDNA gene sequence was analyzed by UCLUST against the Silva (Release119 http://www.arb-silva.de) and Greengene (Release 13.5 http://greengenes.secondgenome.com/) 16S rRNA database using confidence threshold of 90%. Finally, an OTU table was generated for abundance, bacterial composition and further analysis.

1. Fourier transform infrared spectroscopy (FTIR) of BP

BP was ground and mixed with KBr in the ratio of 1:100. With KBr as the reference, the absorption wavelength of BP was measured by Fourier infrared spectrometer in the range of 400−4000 cm^-1^ (23 ± 2 °C). The specimen was obtained by compression of mixed BO and KBr powders (1:100, w/w).

### Supplementary Figures


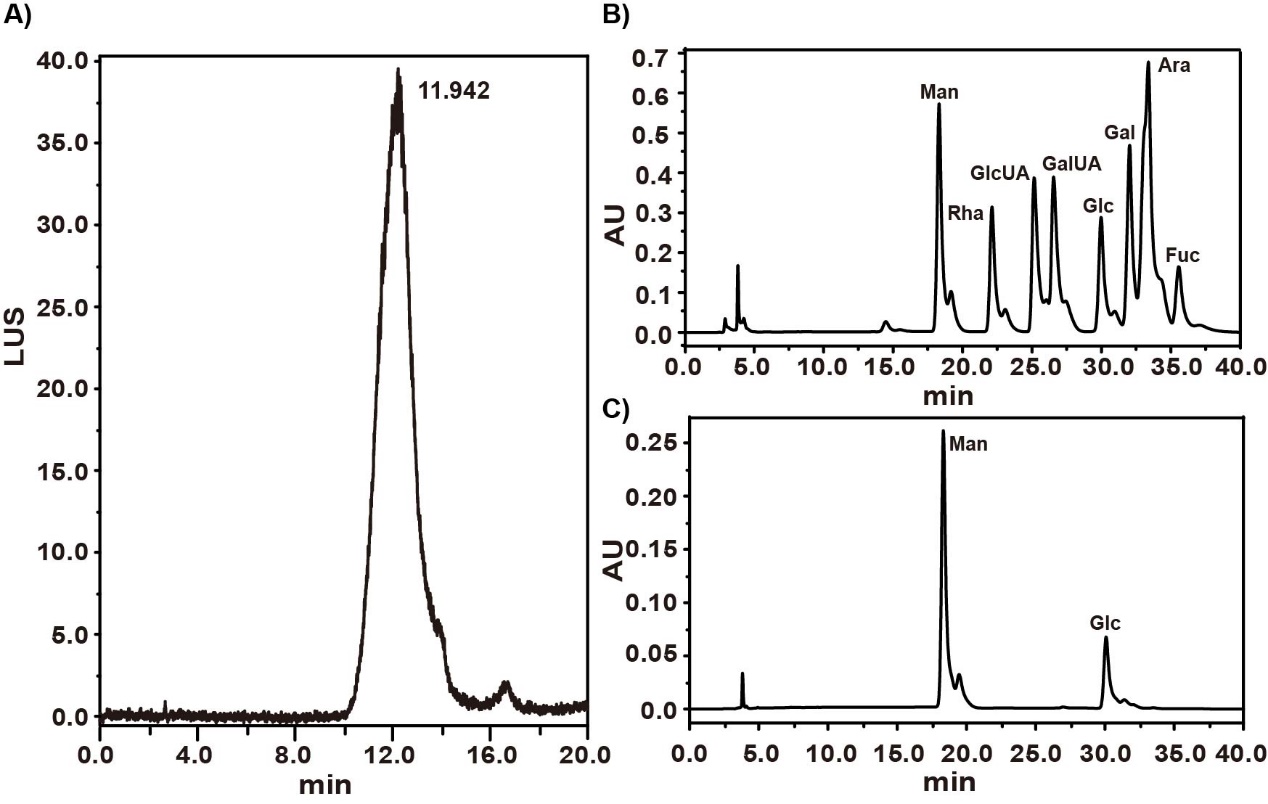


Fig. 1. Chromatographic analysis of BP. (A) Molecular weight assay of BP by high-performance gel permeation chromatography (HPGPC). (B) Chromatogram of monosaccharide standards by high-performance liquid chromatography (HPLC). (C) Monosaccharide composition of BP by HPLC. Man, mannose; Rha, rhamnose; GlcUA, glucose-uronic acid; GalUA, galactose-uronic; Glc, glucose; Gal: galactose; Ara, arabinose; Fuc, fucose.


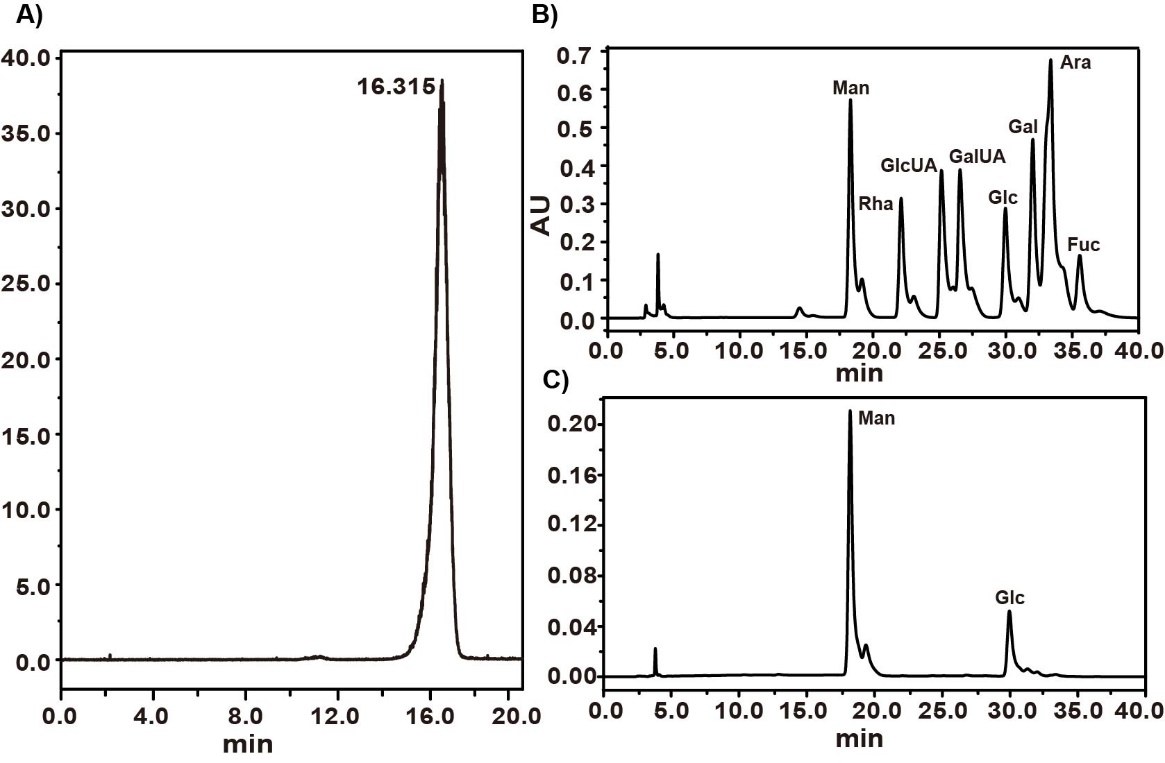


Fig. 2. Chromatographic analysis of BO. (A) Molecular weight assay of BO by high-performance gel permeation chromatography (HPGPC). (B) Chromatogram of monosaccharide standards. (C) Monosaccharide composition of BO by HPLC. Man, mannose; Rha, rhamnose; GlcUA, glucose-uronic acid; GalUA, galactose-uronic; Glc, glucose; Gal: galactose; Ara, arabinose; Fuc, fucose.


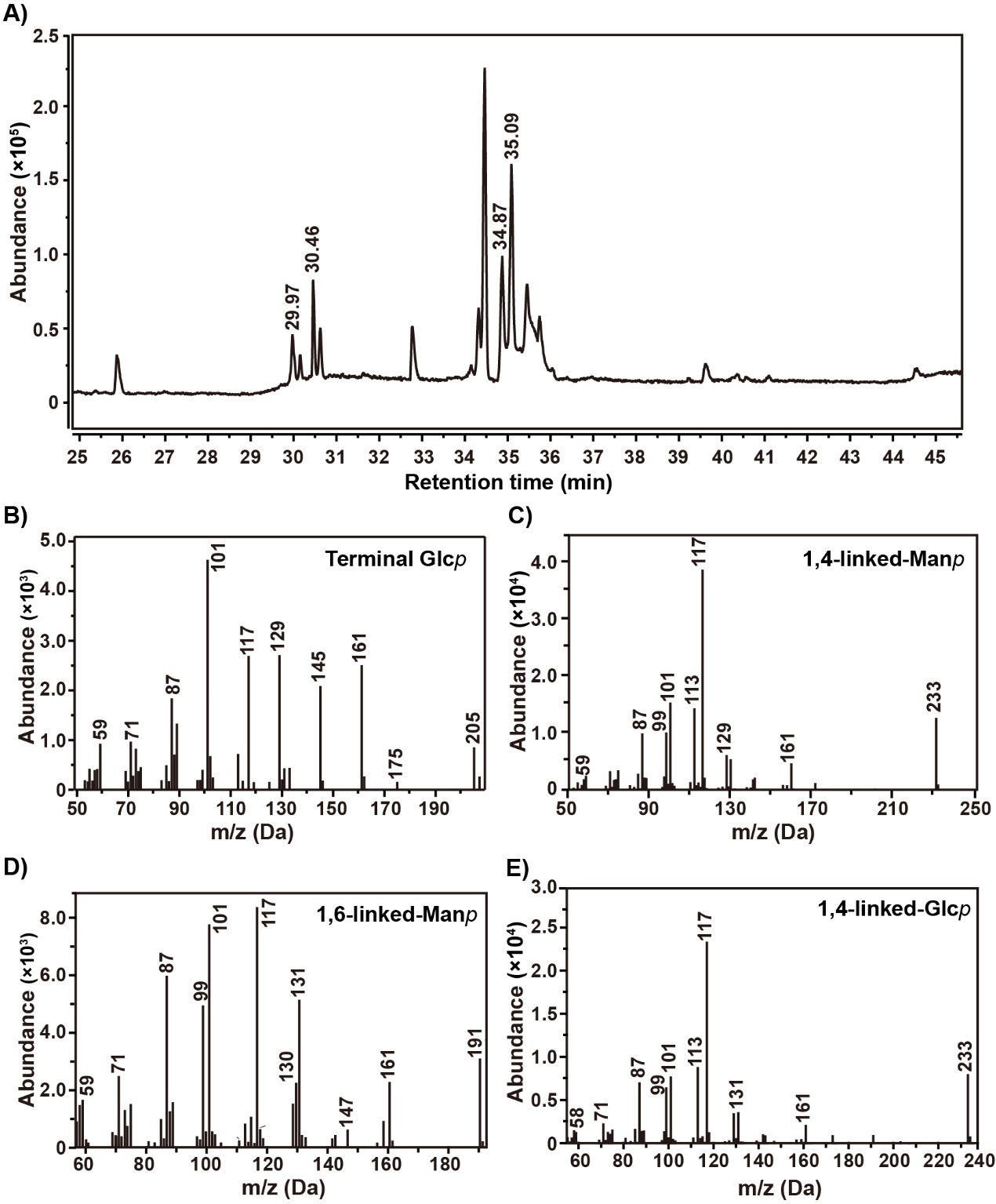


Fig. 3. GC-MS profile of methylated alditol acetates of BP. (A) Total iron chromatogram of BP. (B-E) MS fragments and deduced residues of BP, including Terminal Glc*p* (B), 1,4-linked-Man*p* (C), 1,6-linked-Man*p* (D), and 1,4-linked-Glc*p* (E).


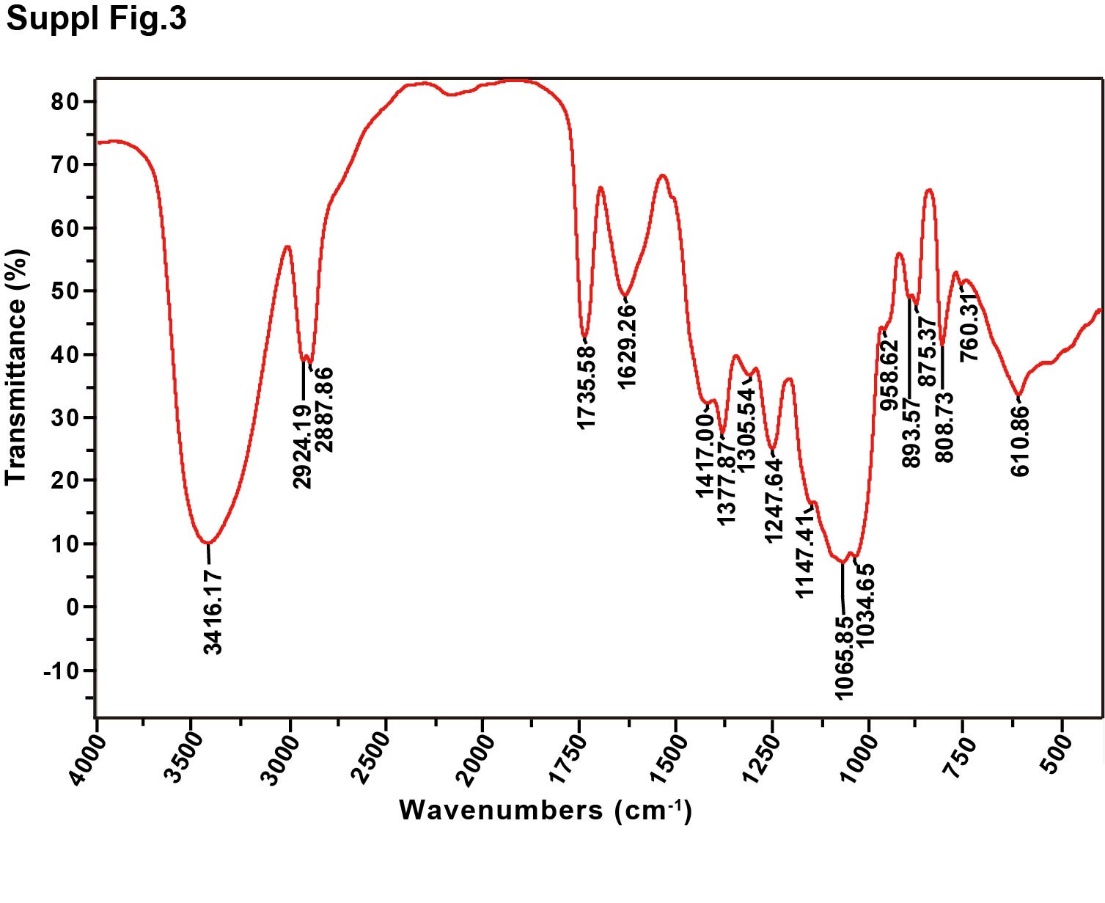


Fig. 4. FTIR spectra analysis of BP.


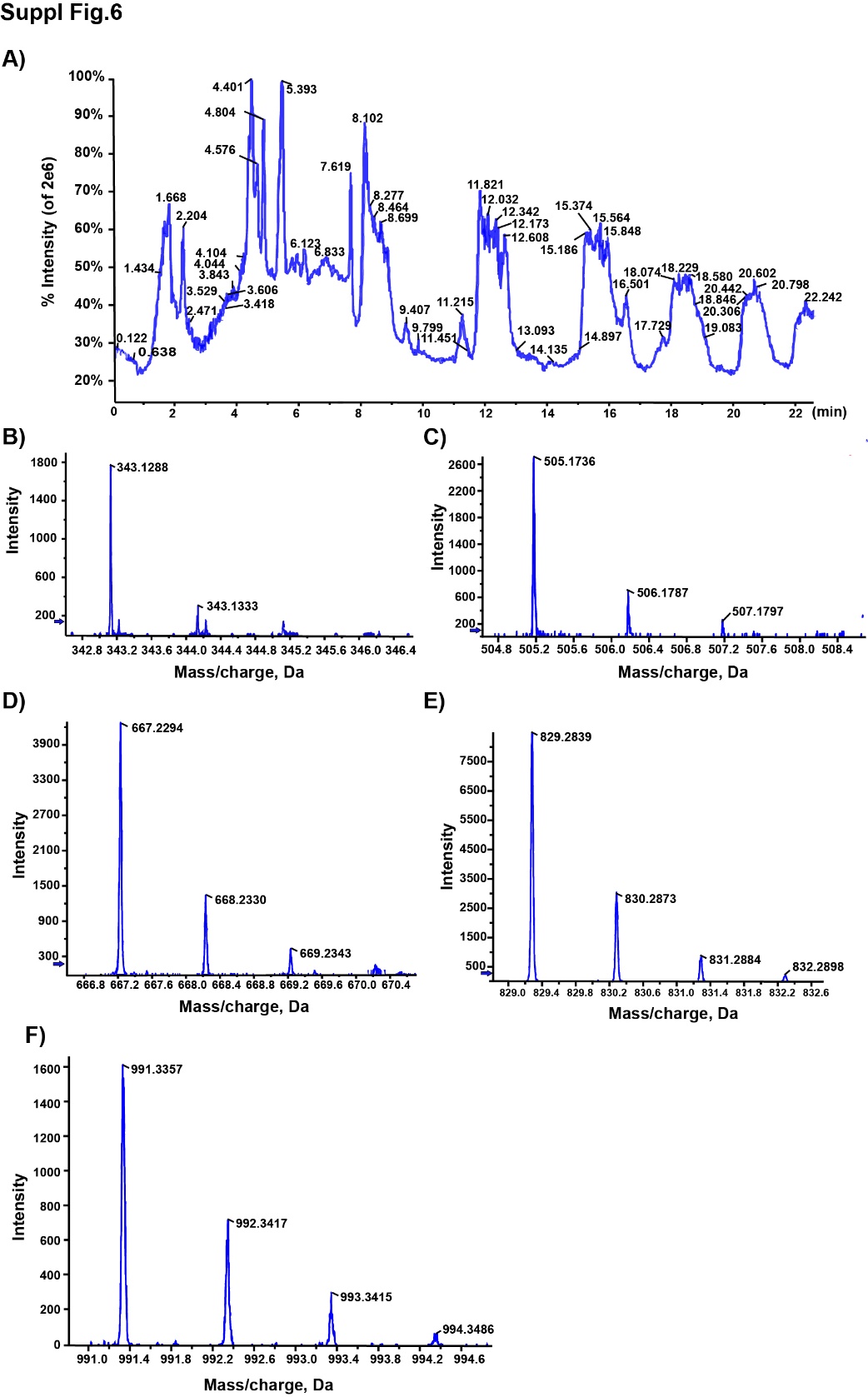


Fig. 5. Structure analysis of BO. (A) Mass spectrogram of BO. (B) Disaccharide mass spectra of BO. (C) Trisaccharide mass spectra of BO. (D) Tetrasccharide mass spectra of BO. (E) Pentasaccharide mass spectra of BO. (F) Hexasaccharide mass spectra of BO.


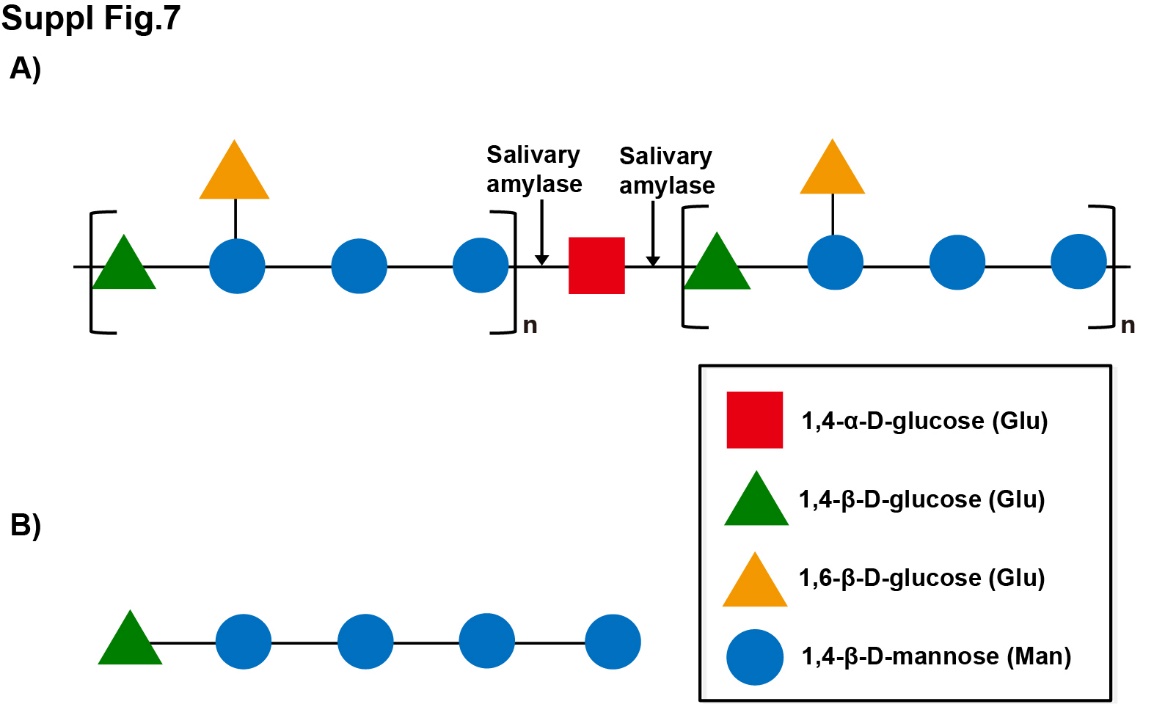


Fig. 6. Predicted structures of BP (A) and BO (B).


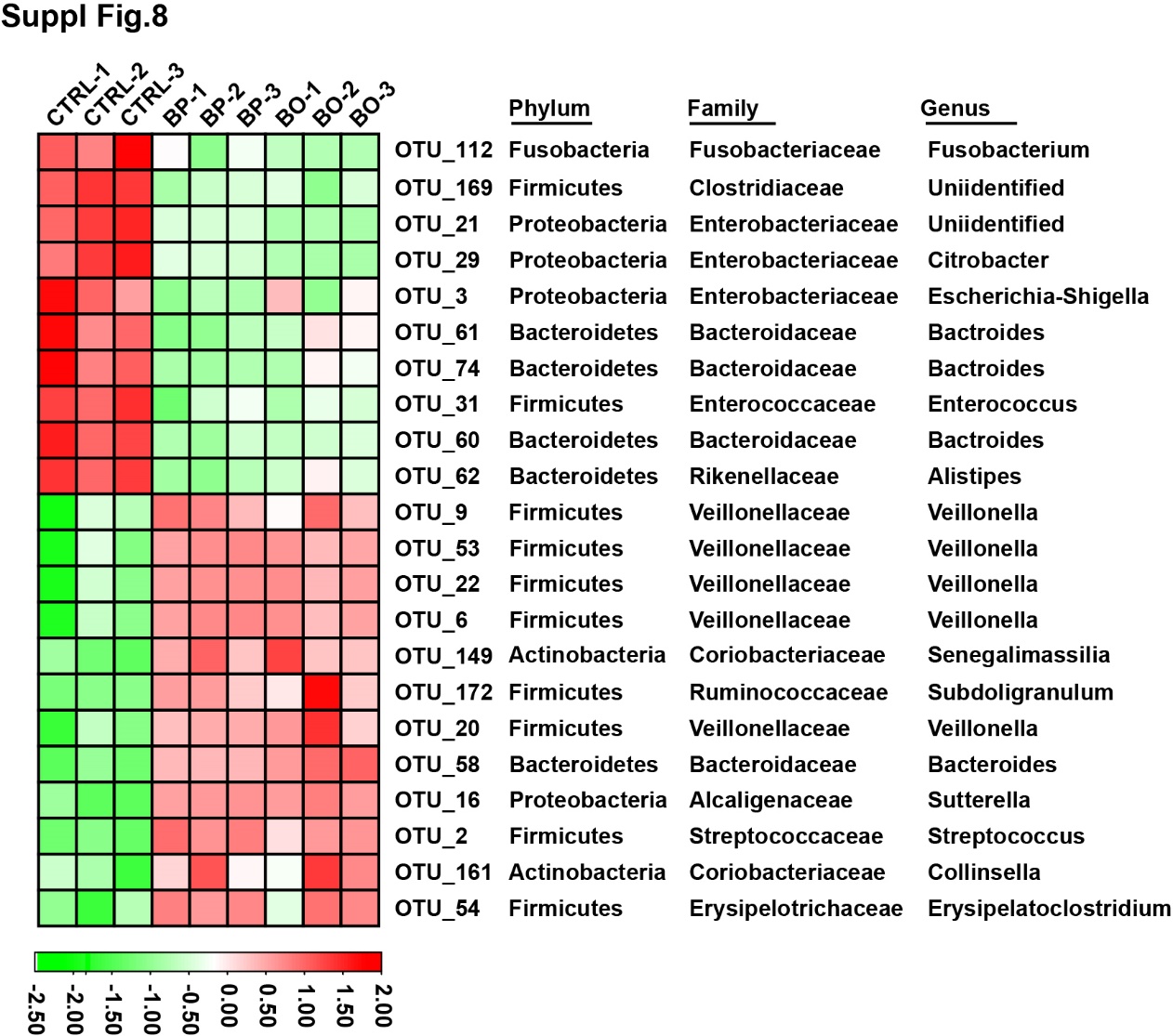


Fig. 7. Heatmap analysis of gut microbiota at genus level using LEfSe analysis.

### Supplementary Tables

Table 1. Sugar residue linkages of BP by methylation and GC-MS

| Retention time (min) | Methylated sugar^a^ | Type of linkage^b^ | Molar ratio (%) | Mass fragments (m/z) |
| --- | --- | --- | --- | --- |
| 29.97 | 2,3,4,6-Me_4_-Glc*p* | Terminal Glc*p* | 7.22 | 59, 71, 87, 101, 117, 129, 145, 161, 175, 205 |
| 30.46 | 2,3,6-Me_3_-Man*p* | 1,4-linked-Man*p* | 47.75 | 59, 87, 99, 101,113, 117, 129, 161, 233 |
| 34.87 | 2,3,4-Me_3_-Man*p* | 1,6-linked-Man*p* | 16.99 | 59, 71, 87, 99, 101, 117, 130, 131, 147,161, 191 |
| 35.09 | 2,3,6-Me_3_-Glc*p* | 1,4-linked-Glc*p* | 28.04 | 58, 71, 87, 99, 101, 113, 117, 131, 161, 233 |

^a^ Analyzed by GC–MS, after per-O-methylation, total acid hydrolysis, reduction, and acetylation.

^b^ Based on derived O-methylalditol acetates.

Table 2. HPGPC analysis exhibiting molecular weight (MW) changes of BP during *in vitro* digestion.

| Types of digestion | Time | HPGPC | | |
| --- | --- | --- | --- | --- |
|  |  | Retention time  (min) | Lg (MW) | MW |
| Salivary digestion | 0min | 12.736 | 5.31 | 202389 |
|  | 5min | 13.834 | 4.44 | 27524 |
|  | 15min | 13.936 | 4.36 | 22867 |
|  | 30min | 13.982 | 4.32 | 21034 |
| Gastric digestion | 0 h | 13.960 | 4.34 | 21892 |
|  | 1 h | 13.950 | 4.35 | 22293 |
|  | 3 h | 13.933 | 4.36 | 22292 |
|  | 5 h | 13.972 | 4.33 | 21419 |
| Intestinal digestion | 0 h | 13.996 | 4.31 | 20505 |
|  | 0.5 h | 14.045 | 4.27 | 18759 |
|  | 1 h | 14.058 | 4.26 | 18321 |
|  | 2 h | 14.041 | 4.28 | 18895 |
|  | 4 h | 14.037 | 4.28 | 19033 |
|  | 6 h | 14.019 | 4.29 | 19666 |

Table 3. HPGPC analysis exhibiting molecular weight (MW) changes of BP and BO during *in vitro* fermentation.

| Types of digestion | Time/h | HPGPC | | |
| --- | --- | --- | --- | --- |
|  |  | Retention time  (min) | Lg (MW) | MW |
| BP | 0 | 11.942 | 5.34 | 221173 |
|  | 6 | 13.300 | 4.63 | 42432 |
|  | 12 | 14.900 | 3.75 | 5561 |
|  | 24 | 15.385 | 3.48 | 3004 |
|  | 36 | 15.378 | 3.48 | 3031 |
|  | 48 | 15.383 | 3.48 | 3011 |
| BO | 0 | 16.300 | 2.97 | 940 |
|  | 6 | 16.300 | 2.97 | 940 |
|  | 12 | 16.300 | 2.97 | 940 |
|  | 24 | 16.300 | 2.97 | 940 |
|  | 36 | 16.300 | 2.97 | 940 |
|  | 48 | 16.300 | 2.97 | 940 |

Table 4. Statistics on raw/clean reads by 16S rDNA sequencing

| Sample ID | Raw_tags | Clean_tags |
| --- | --- | --- |
| Ctrl1 | 49571 | 41035 |
| Ctrl2 | 48633 | 40742 |
| Ctrl3 | 46964 | 39550 |
| BP1 | 35799 | 30633 |
| BP2 | 365323 | 31522 |
| BP3 | 27588 | 23695 |
| BO1 | 46500 | 38853 |
| BO2 | 46955 | 39124 |
| BO3 | 38672 | 31830 |

Table 5. Number of OUTs for single sample

| Sample ID | Final_tags | OTUs |
| --- | --- | --- |
| Ctrl1 | 21649 | 98 |
| Ctrl 2 | 21649 | 95 |
| Ctrl 3 | 21649 | 102 |
| BP1 | 21649 | 99 |
| BP2 | 21649 | 100 |
| BP3 | 21649 | 99 |
| BO1 | 21649 | 105 |
| BO2 | 21649 | 124 |
| BO3 | 21649 | 99 |

Table 6. List of primer sequences for qPCR analysis of fecal samples

| Bacteria | Forward primer (5'-3') | Reverse primer (5'-3') |
| --- | --- | --- |
| *Veillonella* | A(C/T)CAACCTGCCCTTCAG | CGTCCCGATTAACAGAGCTT |
| *Enterococcus* | CCCTTATTGTTAGTTGCCATCATT | ACTCGTTGTACTTCCCATTGT |
| *Escherichia* | AGATGCCCTCGGTCTTTGT | GAGTAATTGATGAGCGTGCTG |
| *Streptococcus* | GTACAGTTGCTTCAGGACGTATC | ACGTTCGATTTCATCACGTT |
